# Young adults in motor vehicle collisions in Norway: user safety errors observed in majority of cases with severe or fatal injuries

**DOI:** 10.1007/s12024-022-00541-x

**Published:** 2022-10-17

**Authors:** Jan Mario Breen, Pål Aksel Næss, Trond Boye Hansen, Christine Gaarder, Harald Klemmetsen, Arne Stray-Pedersen

**Affiliations:** 1 Department of Forensic Sciences, Oslo University Hospital, Division of Laboratory Medicine, P.O. Box 4950, Nydalen, 0424 Oslo, Norway; 2 Department of Traumatology, Oslo University Hospital, P.O. Box 4956, Nydalen, 0424 Oslo, Norway; 3 Division of Prehospital Services, Oslo University Hospital, P.O. Box 4956, Nydalen, 0424 Oslo, Norway; 4 Institute of Clinical Medicine, Faculty of Medicine, University of Oslo, P.O. Box 1171, Blindern, 0318 Oslo, Norway; 5 Section for Traffic and Maritime Services, Oslo Police District, P.O. Box 2094, Vika, 0125 Oslo, Norway

**Keywords:** Motor vehicle collision, Collision investigation, Injury mechanism, Young adults, Safety errors

## Abstract

**Purpose:**

We performed a multidisciplinary investigation of young adults involved in motor vehicle collisions (MVCs) to elucidate injury mechanisms and the role of passive safety equipment such as seat belts and airbags.

**Methods:**

MVCs resulting in death or serious injuries to the driver or passengers aged 16–24 years in southeastern Norway during 2013–2016 were investigated upon informed consent. We assessed the crash scene, the motor vehicle (MV) interior and exterior, and analyzed data from medical records, forensic autopsies and reports from police and civil road authorities.

**Results:**

This study included 229 young adult occupants involved in 212 MVCs. The Maximum Abbreviated Injury Scale (MAIS) score was ≥2 in 111 occupants, of which 22 were fatalities. In 59% (65/111) of the cases with MAIS score ≥2 injuries, safety errors and occupant protection inadequacies were considered to have contributed to the injury outcome. Common errors were seatbelt non-use and misuse, carrying insecure luggage, and the seat back being too reclined. MAIS score ≥2 head/neck injuries were observed in side impacts despite correct seatbelt use, related to older MVs lacking side airbag curtains. The independent risk factors for MAIS score ≥2 injuries included not using a seatbelt, driving under the influence of alcohol or drugs, nighttime driving, side impacts, heavy collision partner, and MV deformation.

**Conclusion:**

User safety errors (not using a seatbelt, seatbelt misuse, excessive seat-back reclining, and insecure cargo) and a lack of occupant protection in older MVs resulted in young adults sustaining severe or fatal injuries in MVCs.

**Supplementary Information:**

The online version contains supplementary material available at 10.1007/s12024-022-00541-x.

## Introduction

Major enhancements in road infrastructure, vehicle safety, and the enforcement of traffic laws have substantially improved road safety in developed countries [1]. However, young drivers have been shown to have the highest crash risk and injury rates on the road [2–4]. In Norway, the rate of road traffic deaths has decreased markedly from approximately 13 to 2.5 per 100,000 persons/year over the last 50 years, which also reflects substantial improvements in the quality of prehospital and hospital care. However, road traffic injuries remain a public health concern, especially for the young population. From 2000 to 2020, 24% of the fatalities and 26% of severe injuries in road traffic incidents in Norway involved young adults aged 16–24 years. Approximately 71% of these victims were injured in motor vehicle collisions (MVCs) and were either drivers or passengers in four-wheeled cars [5].

The Norwegian government has implemented the Vision Zero road traffic safety project as the basis for national traffic safety activities [6], with the ambition of no one being killed or permanently disabled due to road traffic collisions in the future. This impressive goal requires targeted preventive action based on updated knowledge. However, few studies have been dedicated to understanding and investigating the factors underlying the injury severity for young occupants involved in MVCs [7]. Knowledge about what causes a collision and the specific injury mechanisms for occupants is essential. As the limits of human tolerance to impact forces is a central concept of Vision Zero, it is also essential to understand what protects occupants from being injured in a severe collision. Such understanding can lead to designing well-targeted and restrictive measures, and provide the groundwork for political solutions to enhance road safety for young adults.

Based on the aforementioned background, we designed a study focusing on young adults involved in major MVCs on Norwegian roads. We set out to identify the characteristics of a collision leading to injury, including the occupants’ movements inside the motor vehicle (MV) at the time of the collision, and the use and effects of seatbelts, airbags, and other safety measures. By characterizing the injuries sustained by the occupants and the underlying mechanisms, the goal was to determine which injuries are preventable.

## Material and methods

### Investigation protocol

This was a study of young adult occupants involved in high-energy MVCs collected from March 2013 to March 2016 in southeastern Norway. Overviews of the investigation protocol have been published previously [8, 9].

The criteria for inclusion were the occurrence of a high-energy MVC involving one or more occupants aged 16–24 years and at least one of the occupants in the MV being met by a trauma team in the admitting hospital. The investigation team was then alerted by the regional Emergency Medical Communication Centers (EMCCs). The team of investigators started the process of collecting data and was dispatched to the crash scene within 24 h of the collision to investigate the case MV in which the injured young adult had been sitting in. In brief, the collision investigators systematically assessed the exterior and interior environments of the MV, approached the involved occupants to ask them to participate in the study, and after receiving their consent performed an interview. Information was also obtained from emergency medical service personnel, staff from police and civil road authorities, and witnesses where available. Reports on the aftermath of the collision were collected from the EMCC records, police, and the collision-analysis groups of the Norwegian Public Roads Administration. The crash data, including information about road infrastructure and conditions, involved vehicles, and occupants, were collected and categorized in the dataset. With consent from participants or the next of kin, the medical records and autopsy reports were also retrieved.

The internal evaluation of the MV focused on contact points between the occupant and the MV interior (as documented by dents/deformations on the interior surfaces or deposited biological material such as blood, skin, or hair), use of seatbelts (friction marks thereon), safety equipment (number of front airbags, side airbags and side curtain airbags in the MV, airbag deployment, seatbelt pretensioners, and load limiters), and the presence of heavy insecure objects. The front and rear seat backs were inspected to determine whether seats had been displaced or damaged/distorted by the movements of heavy insecure objects. Any such displacement was measured and recorded in centimeters. An assessment of the passenger compartment’s integrity and measurements of component intrusions were also performed during the interior inspection.

The investigators subsequently performed a reconstruction of each occupant’s movements during the collision based on the information obtained from the examination of the MV’s exterior and interior examination and knowledge about the injuries that occurred. A person of approximately the same size was placed in the crashed MV in an attempt to determine the occupant’s movements and possible contact points within the MV’s interior. The reconstruction and technical investigations of the MV exterior, interior, and collision environment were documented using photographs, and a detailed collision report for each MVC was generated by the collision investigators.

Finally, all cases were reviewed by a multidisciplinary team consisting of physicians with expertise in traumatology and forensic medicine, and paramedics with expertise in collision investigations.

### Calculation of collision speed and impacting forces

The principal direction of impact force and the instantaneous change in velocity (Δ*V*) were calculated manually. We superimposed the face of a compass over the illustration of an MV, with 0° (or 360°) aligned at its front. Collision impacts were then categorized into frontal (0 to 45°, 315 to 360°), side (226 to 314° on the left, 46 to 134° on the right), rear (135 to 225°), and rollover (at least 180° on the horizontal axis). The occurrence of an impact did not mutually exclude another impact. Side impacts were further categorized into nearside and offside based on the occupant’s position relative to the collision side.

MV types were classified into passenger car (station wagon, hatchback, or sedan), sport utility vehicle/minibus/minivan, and truck/bus.

### Injury severity

The incident was considered fatal if death was due to injuries sustained during the collision and occurred within the first 30 days thereafter. The Abbreviated Injury Scale (MAIS) score, Maximum MAIS score, Injury Severity Score, and New Injury Severity Score were calculated for each case [10]. Data on the sex, height, and BMI of each occupant were also recorded.

We defined an occupant with an MAIS score of ≥2 in at least one body region as injured**,** while the others were coded as not injured (i.e., had MAIS score = 1 or 0).

### Evaluation of safety errors and injury mechanisms

The likely injury mechanism and the significance of safety errors on the injury outcome for the involved young occupants were determined; that is, whether the injury severity might have been influenced by seatbelt use or misuse, MV crashworthiness, and the presence or absence of passive safety equipment in the case MV. A case-by-case review was performed to evaluate whether safety errors or the failure or absence of safety equipment might have contributed to the mechanism and the extent of injuries.

We defined that an occupant was unrestrained if they were not using any part of the seatbelt. Any incorrect use of the seatbelt (e.g., incorrect routing of the seatbelt under an arm or behind the back, improper twisting of the seatbelt, seatbelt being too loose across the hip or shoulder, or use of a lap belt only) were classified as seatbelt misuse. Improper sitting posture was classified as occupants sitting with their legs on the dashboard, leaning backward or forward while asleep, or having the headrest too low relative to the neck.

The presence of any insecure objects regarded as a potential safety hazard, including unrestrained fellow occupants, was also registered.

### Toxicology

We obtained information on consumption of alcohol, medicinal and illicit drugs from hospital records or police reports. Unfortunately, blood tests were not performed consistently. However, medical records included health care takers’ description of observed behavior that indicated obvious signs of alcohol consumption or drug influence, which then lead to suspicion of impairment without blood toxicology. The legal limit for the blood alcohol concentration (BAC) in Norway was reduced from 0.5 to 0.2 g/kg in 2001. In 2012, Norway also introduced per se limits equivalent of BAC of 0.2 g/kg and 0.5 g/kg for 20 nonalcohol drugs [11]. We chose a threshold level for likely impairment of BAC of ≥ 0.5 g/kg for alcohol, and concentrations equivalent to a BAC of ≥ 0.5 for g/kg for psychoactive medicinal substances or illicit drugs. In cases where medical records clearly indicated obvious signs of alcohol consumption or drug influence, but no blood sample was collected, a BAC ≥ 0.5 g/kg for alcohol or equivalent to BAC ≥ 0.5 g/kg for nonalcohol drugs was assumed.

### Data analyses

The independent Student’s *t*-test was used to evaluate differences in continuous variables according to the mean Δ*V* and collision-related characteristics and types of injuries.

We used a generalized estimation equation (GEE) to investigate the effects of possible explanatory variables (categorical and continuous) on the dependent variable of MAIS score ≥2 injuries (yes/no). The case MVs were entered as the subjects in the data set, and the occupant’s identifiers as within-subjects to adjust for possible intra-MV correlations. An exchangeable correlation structure was used in which constant correlations between any two observations within the MV were assumed. Univariable analyses were first performed to determine the effect of each explanatory variable on MAIS score ≥2 injuries, and we recorded those that had a significance level below 5%.

Variables with a significance level below 10% were entered into the multivariable GEE model to investigate their combined effect on MAIS score ≥2 injuries. The quasilikelihood information (QIC) criterion was used to select the model that provided the best fit to the data [12]; that is, the model with the lowest QIC value. We removed the variable with the weakest association in each analysis, and the calculations were rerun until all remaining explanatory variables were statistically significant. Variables that did not significantly predict the outcome were excluded from the final model.

Odds ratios (ORs) were used to explain the importance of the predictor variables; that is, estimating an occupant’s odds of having an MAIS score ≥2 injury. Estimates of the OR were obtained from the final model, and confidence intervals (CIs) were calculated for all OR values. An OR > 1 indicated a higher likelihood of an MAIS score ≥2 injury. All statistical analyses were performed using SPSS statistical software (version 25.0, IBM Corporation, Armonk, NY).

### Ethics

This study was approved by the Regional Committee for Medical and Health Research Ethics and the Norwegian Prosecuting Authority. Informed consent was obtained from all cases studied or their next of kin.

## Results

**Fig. 1:**
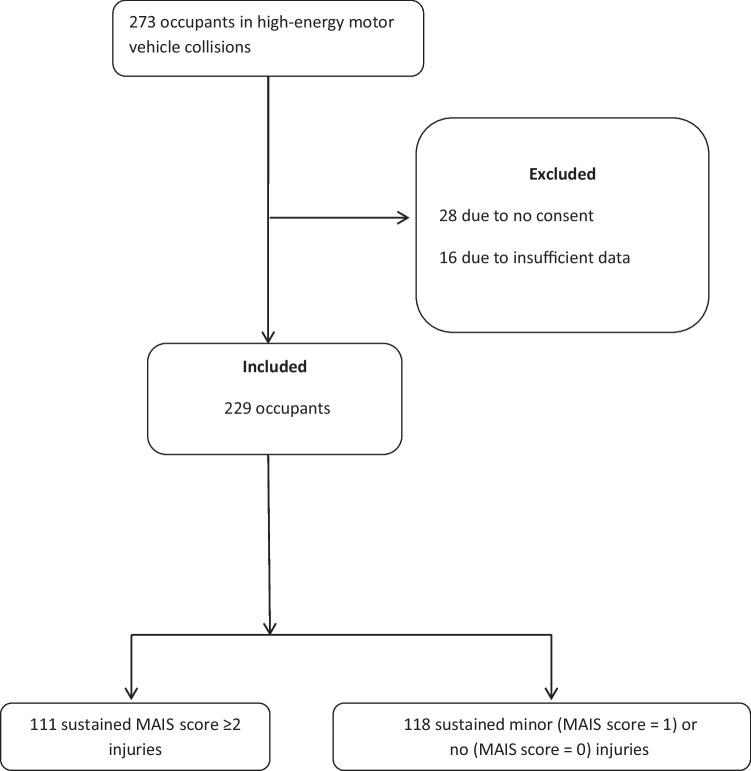
Outline of the study. MAIS, Maximum Abbreviated Injury Scale

During the 3-year study period, we registered 273 occupants involved in high-energy MVCs. Twenty-eight cases (including 15 fatalities) were omitted since we did not obtain their consent to participate, and an additional 16 cases were excluded due to insufficient information on injury outcome, as outlined in Fig. 1.

The demographics of the 229 finally included occupants are presented in Table 1. The median age of the included occupants was 19 years (interquartile range [IQR] = 18–21 years), and 66% (150/229) of them were male. Eight were treated at outpatient clinics only and 221 young adults were admitted to hospital. In total, 111 occupants (48%) sustained 1 or more MAIS score ≥2 injuries in the following body regions: 52 (47%) in the head/face/neck, 59 (53%) in the chest, 33 (30%) in the abdomen, 30 (27%) in the upper extremities, and 42 (37%) in the lower extremities. MAIS score ≥ 3 injuries were observed in 69 cases, and there were 22 fatalities.

**Table 1:** Characteristics of the 229 young adult occupants involved in motor vehicle collisions (MVCs)

**Characteristic**	**Category**	**Occupants with MAIS score ≥2/all occupants (%)**
Sex	Male	74/150 (49)
	Female	37/79 (47)
Age	16–17 years	15/41 (37)
	18–20 years	66/126 (52)
	21–24 years	30/62 (48)
BMI^a^, kg/m^2^	Normal (18.5–24.9)	38/95 (40)
	Overweight/obese (25.0–40.0)	19/36 (53)
Occupant location inside the MV	Left front (driver)	67/128 (52)
	Right front	28/58 (48)
	Left rear	5/16 (31)
	Right rear	10/22 (45)
	Center rear	1/5 (20)
DUI^a^	Yes	28/44 (63)
	No	83/175 (47)
	DUI drivers	19/28 (68)
	DUI front-seat passengers	8/13 (62)
	DUI rear-seat passengers	1/3 (33)
Seatbelt use	Yes	78/178 (44)
	No	33/51 (65)
	Drivers using SB	51/104 (49)
	Front-seat passenger using SB	14/41 (34)
	Rear-seat passengers using SB	13/33 (39)
Number of young adult occupants in MV	1	53/88 (60)
	≥2	58/141 (41)

*MAIS* Maximum Abbreviated Injury Scale, *MV* motor vehicle, *DUI* driving under the influence of alcohol or drugs, *SB* seat belt

^a^Total numbers differ since current information was missing in several cases

The characteristics of the 145 case MVs and the collision-related circumstances are presented in Table 2. Most of the collisions occurred in rural areas (92%), on roads without a barrier between the opposing lanes (98%), and involved passenger cars (85%). Single-MV collision represented more than half of the cases (66%). The median speed for the case MVs at the time of the collision was 80 km/h (IQR = 60–100 km/h), and the median Δ*V* was 51 km/h (IQR = 39–68 km/h).

**Table 2:** Collision characteristics for 145 MVs with 229 young adult occupants involved in MVCs

**Characteristic**	**Category**	**Number of MVs (%)**
**MVs (*n* = 145)**		
MV model years (categories according to introduction of first-, second-, and third-generation airbags)	Pre-1998 (first)	40 (28)
	1998–2006 (second)	77 (53)
	2007–2016 (third)	28 (19)
MV type	Passenger car	125 (86)
	SUV/minibus/minivan	20 (14)
Collision types	Head-on collision between MVs in opposite lanes	49 (34)
	Single-vehicle driving off the road	82 (57)
	Collision between two MVs at an intersection	5 (3)
	Collision between two MVs driving in the same lane	9 (7)
Center barriers between opposing lanes (head-on collisions only, *n* = 49)	Yes	0 (0)
	No	49 (100)
Guardrails present (single-vehicle driving off the road only, *n* = 82)	Yes	1 (1)
	No	81 (99)
Collision partner	Passenger car	34 (23)
	SUV/minibus/minivan	14 (10)
	Truck/bus	14 (10)
	Fixed object (single MVC)	83 (57)
PDOF	Side impact	22 (15)
	Nearside	13 (8)
	Offside	9 (7)
	Frontal impact	83 (57)
	Rear impact	8 (6)
	Rollover	32 (22)
Season	Winter (December to February)	27 (19)
	Spring (March to May)	36 (25)
	Summer (June to August)	48 (33)
	Autumn (September to November)	34 (23)
Time of day	0000 to 0600 h	48 (33)
	0600 to 1200 h	20 (14)
	1200 to 1800 h	45 (31)
	1800 to 2400 h	32 (22)
Weekend	Yes	60 (41)
	No	85 (59)
Road topography	Straight	62 (43)
	Curved	83 (57)
Road visibility	Poor	12 (8)
	Good	133 (92)
Light conditions	Twilight or dark	67 (46)
	Daylight or road lighting	145 (54)
Precipitation	Rain/snow	58 (40)
	None	87 (60)
Area	Rural	129 (89)
	Urban	16 (11)
Speed limit	High (≥ 70 km/h)	73 (50)
	Low (< 70 km/h)	72 (50)
Insecure cargo	Yes	43 (30)
	No	102 (70)
Driver’s length of licensure^a^	<1 year	55 (50)
	≥1 year	55 (50)

*SUV* sport utility vehicle, *PDOF* primary direction of force, *MV* motor vehicle

^a^Total numbers differ since current information was missing in several cases

**Fig. 2:**
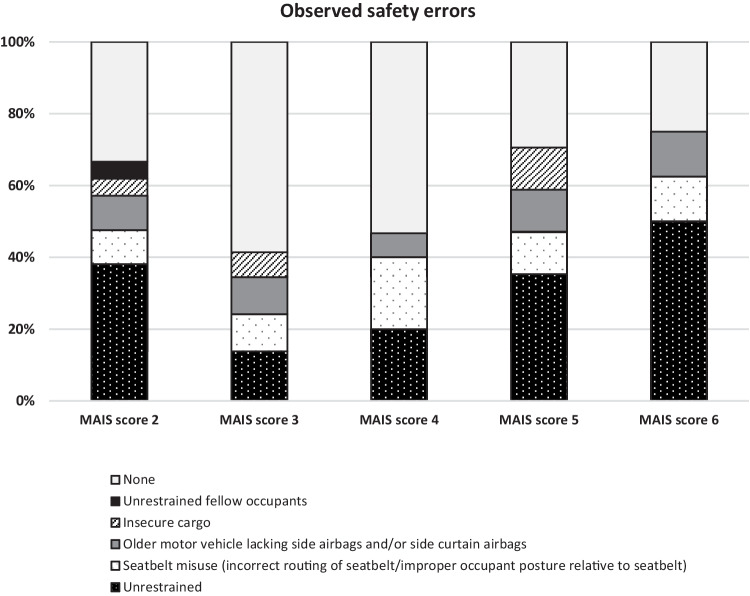
Distribution of safety errors considered contributory to the injury outcome in the 111 occupants with Maximum Abbreviated Injury Scale (MAIS) score ≥2. Safety errors or inadequate occupant protection were observed in 59% (65/111) of the occupants with MAIS score ≥2 injuries, as described in detail in the Appendix (Supplementary Tables 1, 2, 3, 4, and 5). A high proportion of occupants with MAIS score 5 and 6 were either unrestrained, misused seat belts or driving old vehicles with inadequate protection. The proportion of various safety errors according to each MAIS score group was not significantly different

**Fig. 3:**
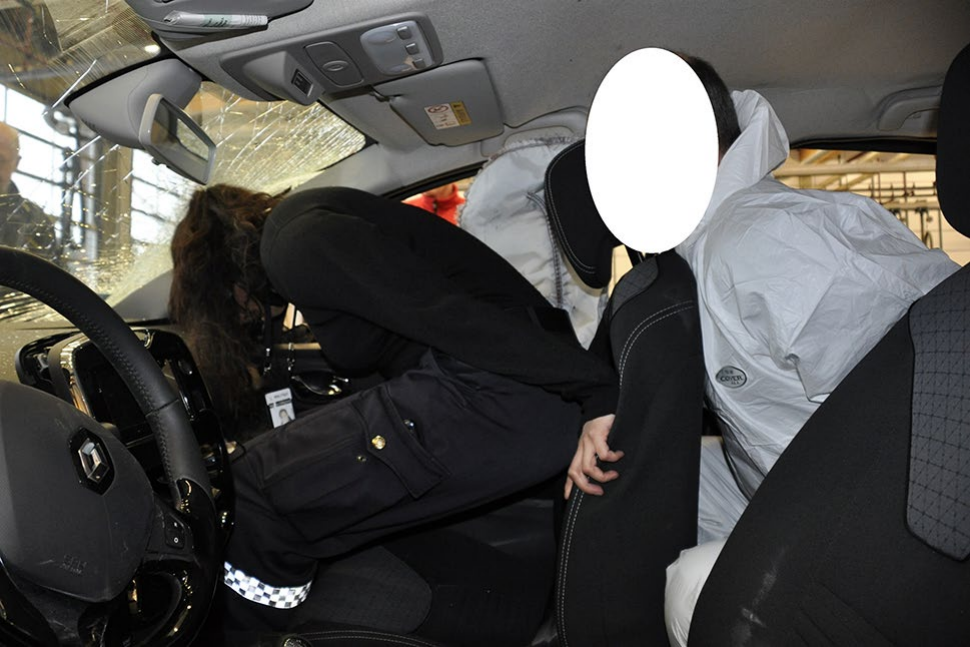
Reconstruction of injury mechanism for front-seat passenger and right rear-seat passenger involved a collision. The main impact was to the right front of the MV. Unrestrained front-seat and unrestrained rear-seat passenger. In addition to no seatbelt use, the forward movement of the rear-seat passenger displaced the front-seat passenger’s seat back forward, thereby increasing the forward momentum and impact load. Injuries: The front-seat passenger sustained fatal head injuries after impact with the windshield/A-pillar and chest injuries after impact with the dashboard

**Fig. 4:**
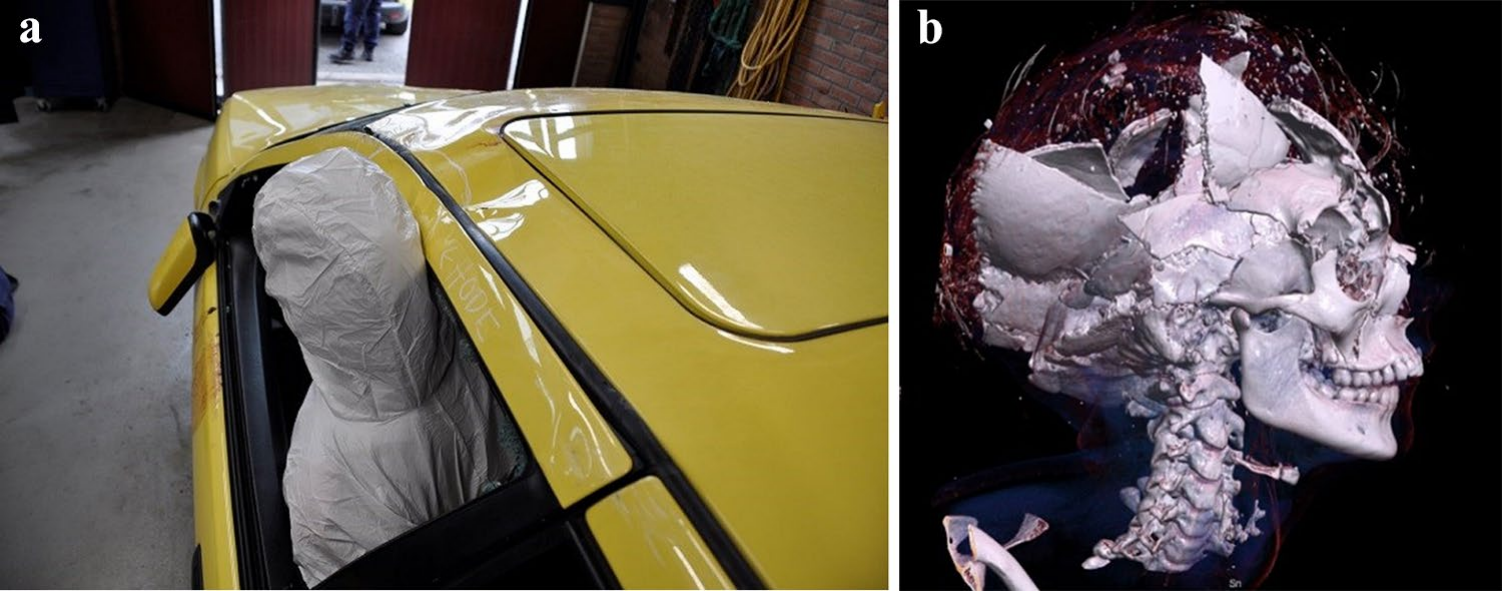
Reconstruction of injury mechanism for driver involved in a fatal rollover incident (**a**). Unrestrained driver was ejected through the side door window during the rollover incident, and the head squeezed between the car roof and the ground. Injury: Fatal head trauma (**b**)

**Fig. 5:**
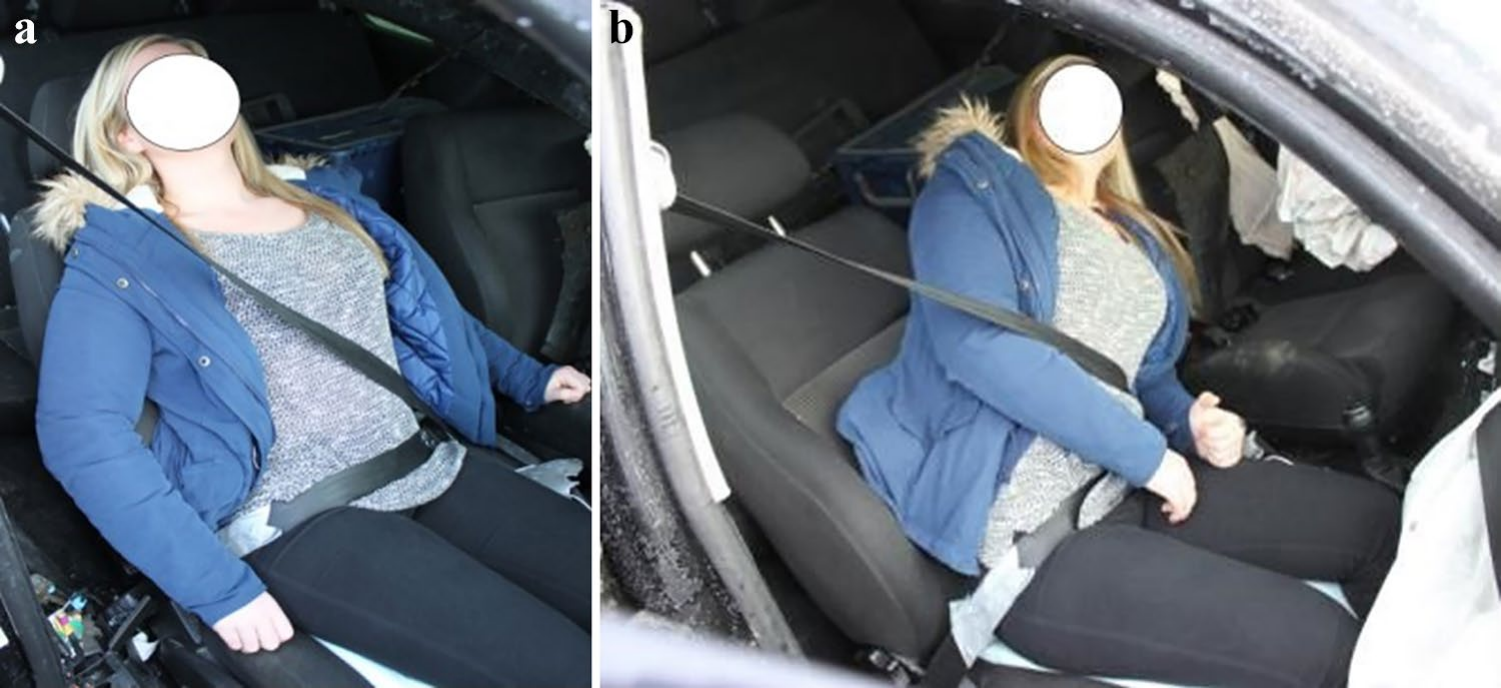
Reconstruction of the injury mechanism for front-seat passenger involved in a collision. The main impact was to the left front of the MV. Seatbelt misuse due to improper occupant posture relative to seatbelt. The seatbelt did not tighten effectively across the occupant’s torso during the collision (**a**). The shoulder part of the seatbelt was too loose due to an overly reclined seat back that allowed for excessive movement of the head and torso forward and to the left (**b**), contributing to direct contact with the driver/driver’s seat back. Injuries: Traumatic head injury (DAI grade 3) and subdural hemorrhage

**Fig. 6:**
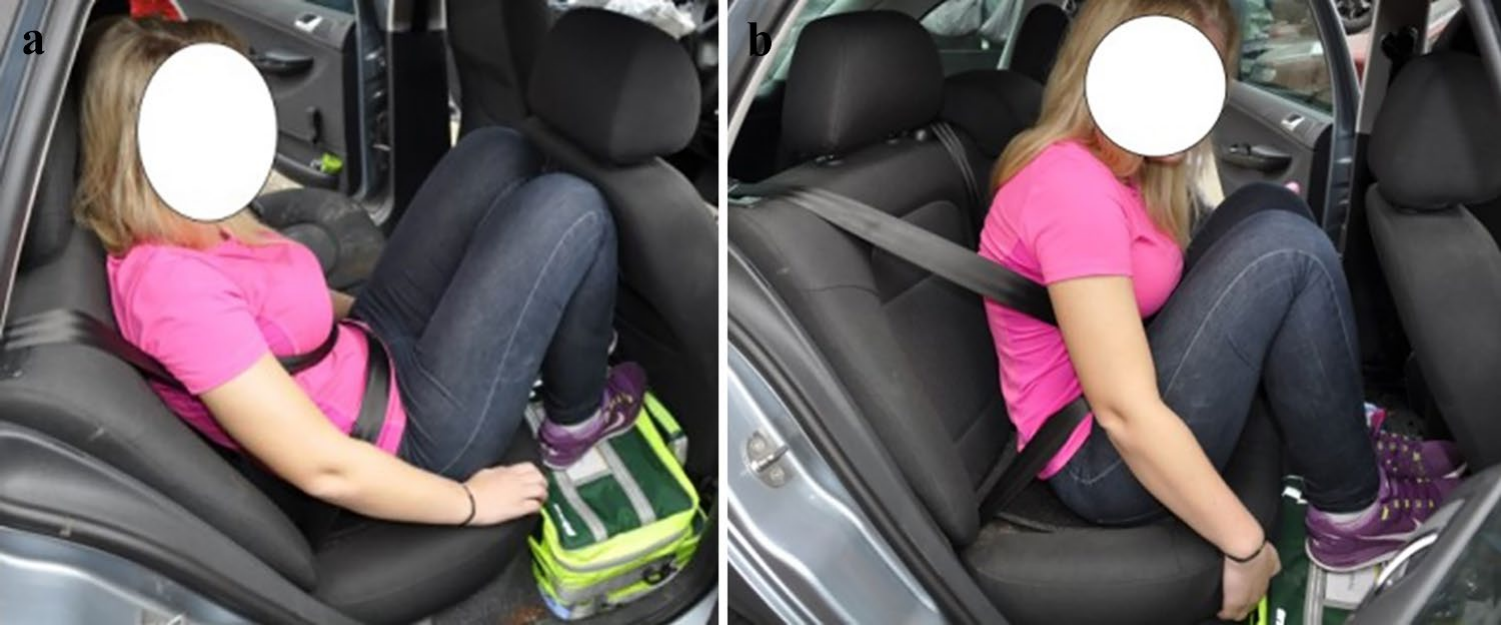
Reconstruction of injury mechanism for right rear-seat passenger involved in a frontal collision. Seatbelt misuse due to incorrect routing of the seatbelt and improper occupant posture relative to the seatbelt. The shoulder part seatbelt was routed under the right arm and the lap belt was too high over the abdomen (**a**). Moreover, the occupant’s legs were on top of luggage placed on the floor behind the rear seat back. The frontal collision caused excessive seatbelt loading directly on abdomen and the right part of the torso (**b**). Injuries: Decollement of abdominal wall, rupture of the diaphragm, herniation and laceration of the gastric ventricle and intestines, bilateral lung contusions, spleen rupture, and major liver injury

**Fig. 7:**
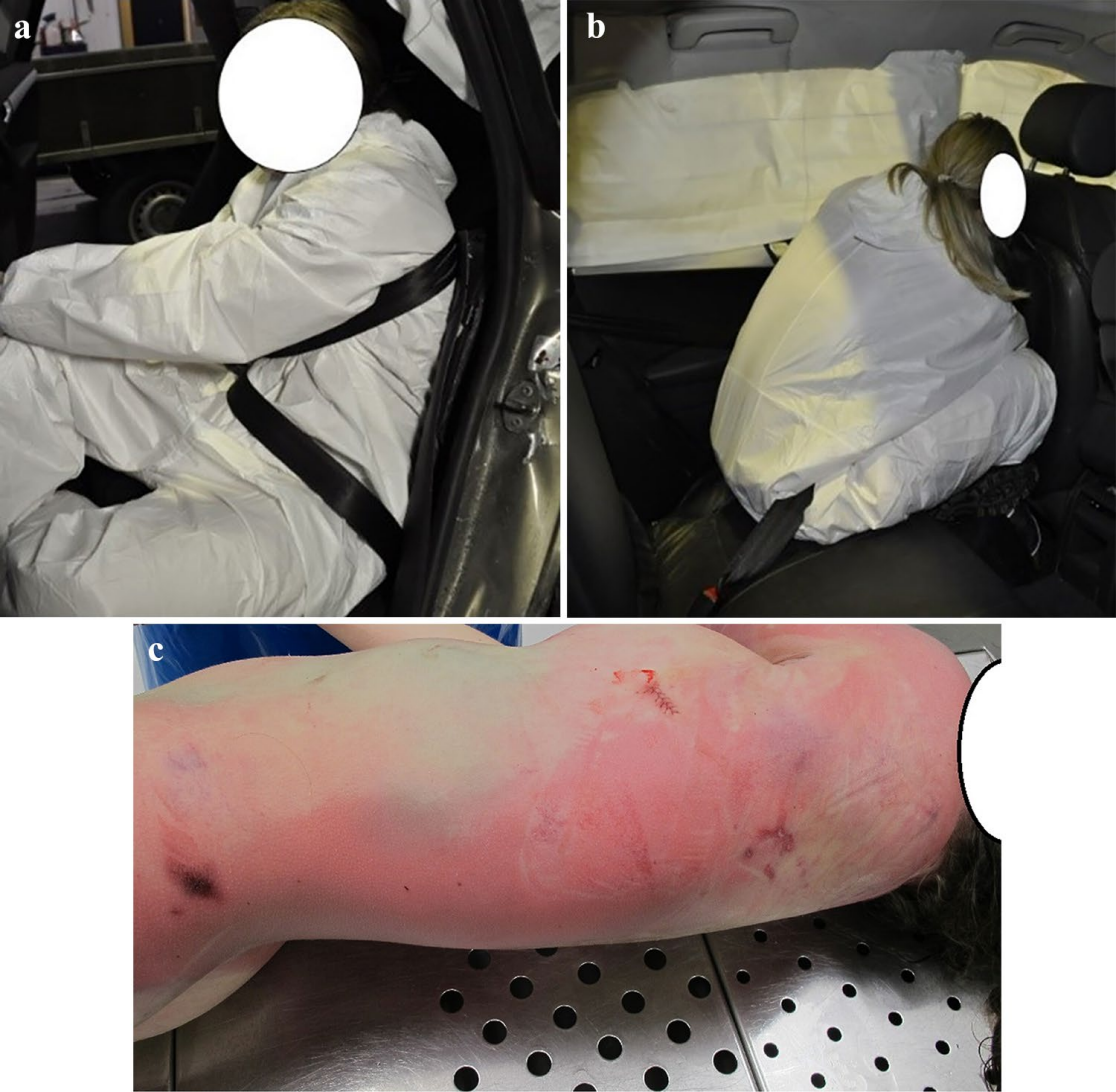
Reconstruction of injury mechanism for left rear-seat passenger involved in a fatal nearside collision (**a**). Seatbelt misuse due to incorrect routing of the shoulder part of the seatbelt under the left arm that allowed for excessive forward movement of the head (**b**) and torso and direct contact with the driver’s seat back. Insecure cargo in the trunk displaced the rear seatback >20 cm forwards, and increased the seatbelt loading on the torso. Injuries: Bruising and abrasion suggestive of seatbelt mark **b**) (**c**) was found under the left arm, as well as mandibular fracture, fatal traumatic brain injury, traumatic subarachnoid hemorrhage, C1-fracture, multiple rib fractures, and a major spleen rupture

**Fig. 8:**
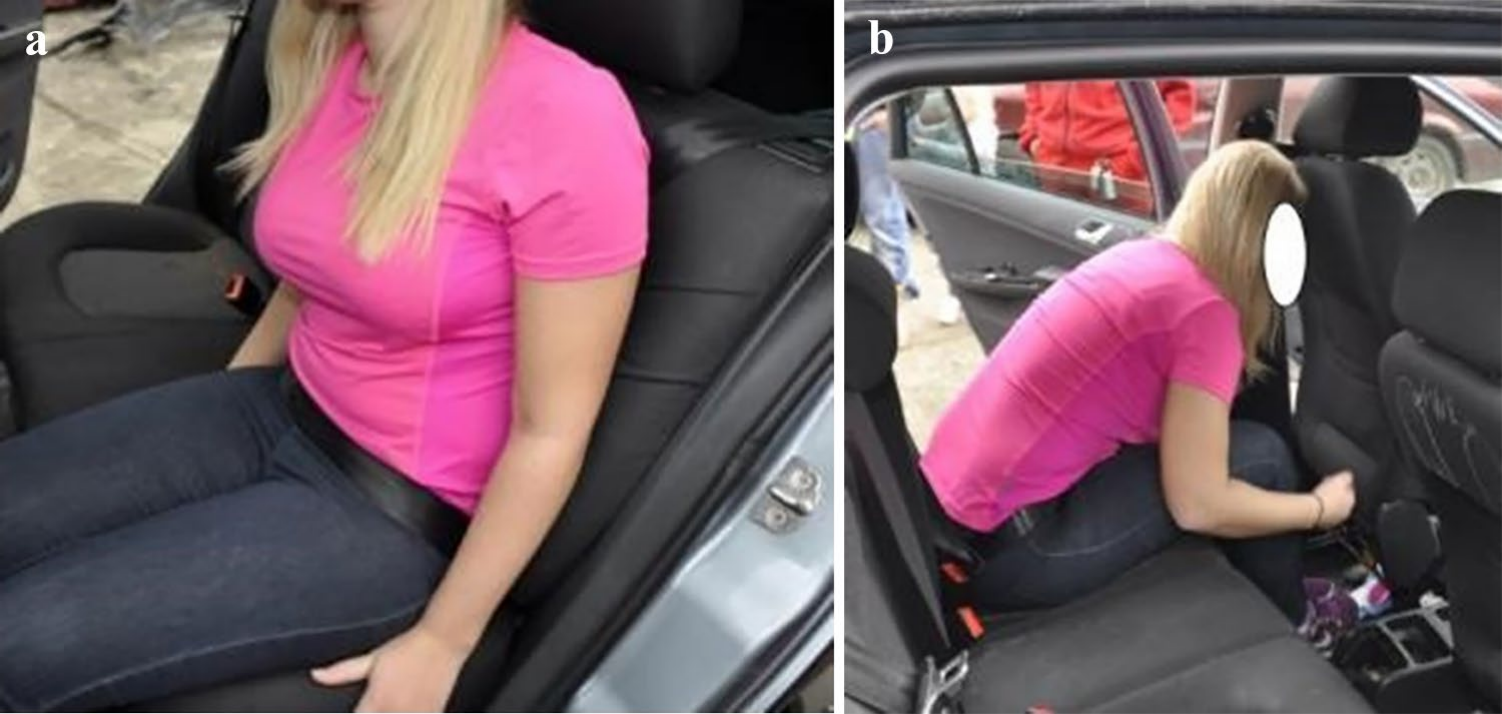
Reconstruction of injury mechanism for left rear-seat passenger involved in a frontal collision. Seatbelt misuse due to incorrect routing of the seatbelt behind the back (**a**) allowing for excessive forward movement of the head and torso and direct contact with the driver seat back, displacing it >10 cm forward (**b**). Injuries: Luckily, only minor head contusion and lung contusions

**Fig. 9:**
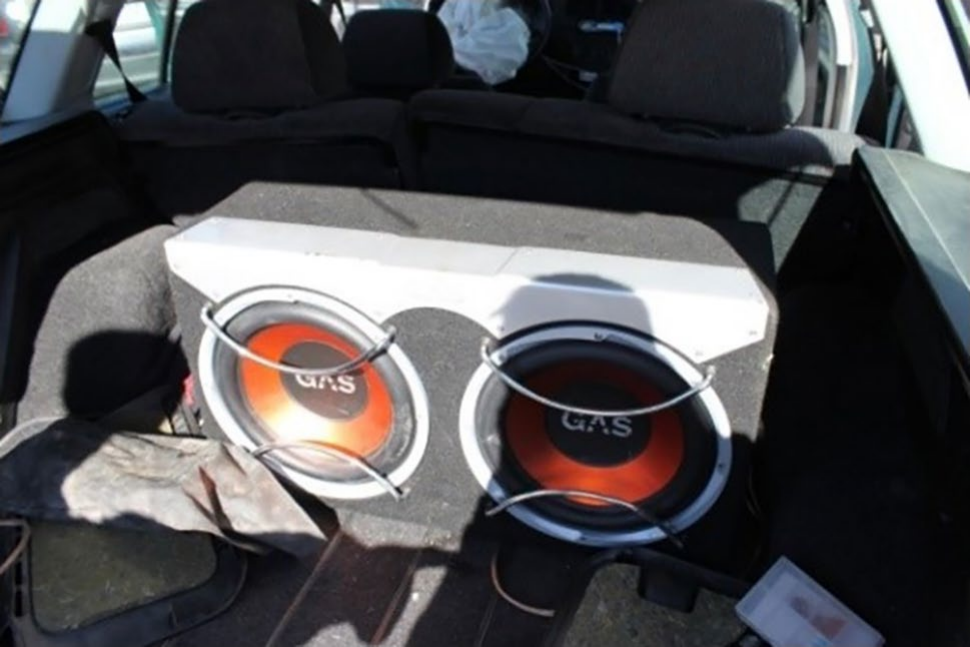
Evidence of insecure heavy cargo in the trunk displacing the rear-seat row forwards in a frontal collision. Luckily, no occupants were sitting in the rear-seat row during the collision

Safety errors or missing standard safety equipment that most likely contributed to the injuries were detected in 59% (65/111) of the occupants with MAIS score ≥2 injuries (Fig. 2). These 65 case characteristics and safety errors observed are described in detail in the Appendix, in Supplementary Tables 1, 2, 3, 4, and 5. In 33 cases, the occupant had been completely unrestrained, thereby exposing them to severe impacts with parts of the MV interior or other occupants, or to being ejected from the MV. There was evidence of a reclined sitting position/occupant posture or seatbelt misuse in 13 cases: 7 with the shoulder part of the seatbelt not in contact with the occupant due to seat back being in an excessively reclined position or the occupant sitting in a slouched position (e.g., sleeping), 5 with the shoulder part of the seatbelt being incorrectly routed under the arm, and 1 with the lap belt being too high over the abdomen. Insecure cargo resulted in secondary impacts to six occupants, and two were impacted by an unrestrained fellow occupant. In 11 cases, the lack of airbag protection was probably of significance, since these occupants all sustained head or neck injuries due to impacting side doors or pillars, which could have been prevented by deploying side airbags or side curtain airbags. Figures 3, 4, 5, 6, 7, 8, and 9 demonstrate safety errors revealed after reconstructions of occupant’s movements inside the MV during the collision.

The multivariable analysis (Table 3) revealed that independent factors for severe injury were related to the occupants, the road environment, and the collision type. The occupant-type and environment-type factors associated with MAIS score ≥2 injuries included not using a seatbelt (OR = 3.28, *p* = 0.009), driving under the influence of alcohol or drugs (DUI) (OR = 2.94, *p* = 0.026), nighttime driving (OR = 2.41, *p* = 0.027), and poor road lighting (OR = 2.50, *p* = 0.043). The collision-type factors associated with MAIS score ≥2 injuries were primary side impacts (OR = 3.38, *p* = 0.015), heavy collision partner (OR = 5.78, *p* = 0.035), and MV deformation (OR = 1.04 for each centimeter increase in deformation, *p* < 0.001). The speed at impact and Δ*V* were both associated with MAIS score ≥2 injuries in the univariable analysis, but did not reach significance in the multivariable model, which was attributable to the correlation with the extent of the MV deformation.

**Table 3:** Collision characteristics predictive of injury. Effects of variables on MAIS score ≥2 injuries in young adult occupants. Predictive values of AIS score ≥2 injuries were calculated using a generalized estimation equation

**Characteristic**	**Univariable analyses of MAIS score ≥2 injuries**			
	**Category or value**	**OR**	**95% CI**	***p***
Sex	Female* vs male	1.12	0.67–1.86	0.672
Age, years	16* to 24	1.06	0.93–1.21	0.389
BMI, kg/m^2^	Normal (18.5–24.9)* vs overweight/obese (25.0–40.0)	1.57	0.71–3.47	0.262
Occupant location inside the MV	Rear* vs front	1.30	0.72–2.34	0.377
DUI	No* vs yes	1.97	1.01–3.86	0.048^a^
Seatbelt use	Yes* vs no	2.11	1.10–4.04	0.024^a^
Young driver alone in the MV	Accompanied by young adult passengers* vs young driver alone	2.12	1.05–4.30	0.037^a^
Occupant protected by front airbags	No* vs yes	1.09	0.65–1.84	0.744
Occupant protected by side airbags	No* vs yes	0.91	0.53–1.56	0.741
Occupant protected by side airbags for head and neck	No* vs yes	0.97	0.50–1.87	0.917
Belted occupant protected by seatbelt pretensioner	No* vs yes	0.97	0.51–1.85	0.922
Belted occupant protected by seatbelt load limiter	No* vs yes	0.61	0.32–1.18	0.142
Belted occupant with “seatbelt sign”	No* vs yes	1.70	0.79–3.66	0.174
**Road type and conditions at time of crash**				
Season	Winter/autumn* vs spring/summer	1.30	0.72–2.35	0.383
Nighttime driving (0000–0600 h)	No* vs yes	2.77	1.42–5.39	0.003^a^
Weekend	No* vs yes	1.26	0.67–2.37	0.466
Road topography	Straight* vs curved	0.82	0.45–1.49	0.514
Road visibility	Good* vs poor	1.59	0.62–4.05	0.334
Barrier between opposing lanes	Yes* vs no	0.61	0.06–6.98	0.695
Light conditions	Daylight/road lighting* vs twilight/dark	1.74	0.92–3.31	0.089
Speed limit	Low (< 70 km/h)* vs high (≥ 70 km/h)	1.55	0.56–2.81	0.145
Precipitation	None* vs rain/snow	1.37	0.67–2.80	0.387
Area	Urban* vs rural	2.21	0.85–5.79	0.105
**MV type and crash characteristics**				
MV age	< 10 years* vs ≥ 10 years	0.78	0.41–1.49	0.446
Driver’s length of licensure	≥ 1 year* vs < 1 year	0.93	0.44–1.97	0.849
Number of MVs	One* vs multiple	0.99	0.55–1.78	0.978
Truck/bus collision partner	No* vs yes	7.11	2.03–24.8	0.002^a^
PDOF				
Side impact	No* vs yes	2.95	1.29–6.71	0.010^a^
Frontal impact	No* vs yes	0.96	0.53–1.73	0.890
Rear impact	No* vs yes	0.48	0.11–2.06	0.323
Rollover	No* vs yes	0.59	0.28–1.21	0.150
Mileage of current trip before crash	≥25 km* vs <25 km	1.13	0.54–2.39	0.742
Driver culpability in the crash	Nonculpable* vs culpable	1.07	0.76–1.49	0.709
Speed at impact	0* to 140 km/h	1.02	0.99–1.02	0.087
Δ*V*	4* to 140 km/h	1.03	1.02–1.05	<0.001^a^
MV deformation (side, front, or rear)	0* to 218 cm	1.03	1.02–1.04	<0.001^a^
Roof deformation	0* to 106 cm	1.02	1.01–1.04	0.030^a^
Purpose of driving trip	Other* vs party-related driving/cruising	1.76	0.71–4.35	0.219

	**Multivariable analyses of MAIS score ≥2 injuries**			
		**OR**	**95% CI**	***P***
DUI	No* vs yes	2.94	1.14–7.56	0.026^a^
Seatbelt use	Yes* vs no	3.28	1.35–8.00	0.009^a^
Truck/bus collision partner	No* vs yes	5.78	1.13–29.7	0.035^a^
Nighttime driving (0000–0600 h)	No* vs yes	2.41	1.11–5.26	0.027^a^
Light conditions	Daylight/road lighting* vs twilight/dark	2.50	1.03–6.05	0.043^a^
PDOF, side impact	No* vs yes	3.38	1.14–9.01	0.015^a^
MV deformation (side, front, or rear)	0* to 218 cm	1.04	1.02–1.05	<0.001^a^

*OR* odds ratio, *CI* confidence interval, Δ*V* instantaneous change in velocity, *DUI* driving under influence, *MV* motor vehicle, *PDOF* primary direction of force, *BMI* body mass index

^*^Reference category

^a^Significantly different (*p* < 0.05) from reference

## Discussion

This study has documented that safety errors and missing safety equipment in the MVs were present in 59% of the cases when young adults sustained MAIS score ≥2 injuries on Norwegian roads. In addition, it was found that many of the injuries and several fatalities could have been prevented by properly restraining and protecting the occupants using airbags.

A high-energy MVC results in the abrupt deceleration and deformation of the MV, exposing the occupants to a diversity of motions and potential impacts. Severe occupant injuries are almost invariably the result of direct contact to the head or chest. The occupant commonly makes contact with certain parts of the MV interior, such as the windshield, roof, or steering wheel. In some cases, the occupant will receive secondary impacts from moving objects within the MV [13]. The complexity of these movements needs to be considered when assessing the effects of various safety measures. Very occasionally there are reports of unrestrained occupants involved in extremely high-energy MVCs who miraculously survive, sometimes even being able to leave the crash scene without any significant injuries. The protective role of seatbelt use against the risk of most major injuries is well established [14], since this often prevents occupants from being thrown into MV structures or completely ejected from the MV [15]. A meta-analysis of 24 studies from 2000 onward found that seatbelt use can reduce fatal and nonfatal injuries the front-seat occupants by 60%, and fatal and nonfatal injuries in rear-seat occupants by 44% [16].

The present study has revealed that a considerable proportion (33/111, 30%) of the young adults who died or were severely injured were not using a seatbelt. The nine fatalities among these all sustained crush injuries due to impacts to the head or chest. The investigations performed in each case showed that a correctly restrained occupant most likely would have survived the impacts, since there was sufficient space to avoid impacts within the MV. We observed that the risk of MAIS score ≥2 injuries was 3.3-fold higher for unrestrained than restrained young adult occupants, after adjusting for other collision-related factors. This analysis also identified that several unrestrained occupants were fortunate to avoid severe injuries.

Why do young adults continue to drive unrestrained? A report from the World Health Organization suggested they do not use seatbelts for reasons such as the driving trip being short, simply forgetting, or being in a hurry [15]. Bad habits and misunderstanding of the safety effects of seatbelts have also been reported [15, 17, 18]. To complicate matters, young adults (and particularly males) have higher crash rates than other age groups [19], which has led to the increased crash risk being attributed to factors such as brain immaturity, susceptibility to high-risk driving behaviors (e.g., speeding), short driving experience, and DUI [2, 9, 20, 21].

DUI was associated with an almost threefold higher risk of severe injuries. Additionally, nighttime driving and driving on dark roads were independent risk factors for injuries with an MAIS score ≥2. Young adult drivers and passengers commonly combine alcohol consumption with reckless driving, speeding, and not using a seatbelt, which is why they have a higher risk of collisions and injuries during nighttime [7, 9, 20, 21]. Williams also suggested that late-night driving increased the crash risk due to driving being more difficult in darkness, particularly for newly licensed drivers [22]. Public transport is scarce at nighttime in rural areas of Norway, leading young people to drive more for social purposes or for pleasure. Possible preventive measures might include restricting young drivers to certain times of the day or, in particular, certain days of the week. Restrictions on nighttime driving and driving with peer teenage passengers have become part of graduated licensing systems in the USA [23] and New Zealand, and they are showing positive effects [7]. An alternative strategy has shown promising results in Norway, where instead of a graduated licensing system, the age limit for supervised practice is 16 years while the licensing age is 18 years, which gives the learner driver an opportunity to acquire more driving experience before being allowed to drive on their own [24]. Moreover, the especially strict Norwegian penalty point system for young drivers (who are punished with double the number of points in the first 2 years after they have obtained their driving license compared with older drivers) has showed promising results in reducing the risk of injury for young adult MV occupants [25].

The multivariable analysis also revealed associations of MAIS score ≥2 injuries with side impacts and MV deformation. Examinations of the MV interiors revealed that the following elements increased the injury severity during side impacts: improper sitting position, insecure cargo or fellow occupants, not using a seatbelt, improperly tightened shoulder part of the seatbelt, and lack of side airbags and side curtain airbags. Moreover, a reclined sitting position at the time of collision suggested that the occupant was not sitting optimally in relation to the door and B-pillar, resulting in the protective effect of side airbags being suboptimal, as described by previously by others [26].

If the shoulder part of the seatbelt is too loose, it will not tighten effectively across the occupant’s torso during a side impact, increasing the risk of excessive body movements and occupant-to-occupant injury. In two cases, we suspected an injury mechanism where the young adult occupant was injured by the intruding structure but also injured by contact with the adjacent occupant in the same seating row who had their shoulder part of the seatbelt too loose. Both Siegel et al. and Hillary et al. suggested an association between side impacts and increased injury severity [27, 28]. Compared with frontal impacts, seatbelts provide reduced protection in side impacts [29, 30], and occupant-to-occupant contact injuries are reportedly more prevalent [31]. Newland et al. also suggested that occupant-to-occupant injury is probably underreported since there is often little evidence in the MV available to investigators of impacts between two occupants. Those authors also demonstrated that drivers having a restrained front-seat passenger present during near-side impacts had an increased risk of MAIS score ≥ 3 injuries, with the risk further increasing if the passenger was not using a seatbelt [32].

The statistical analysis indicated that some variables were not independently predictive of the injury outcome. Some safety errors were present among both those with and without MAIS score ≥2 injuries, but the in-depth analysis still revealed that these errors had detrimental effects on occupant safety. Overall, misuse of seatbelts, unsafe seating positions, and insecure cargo were errors present for 18 occupants who suffered MAIS score ≥2 injuries. Six of them had the shoulder part of the seatbelt incorrectly routed under the arm, which during a collision increased the upper body and head movements and the pressure against the abdomen, resulting in severe head and abdominal injuries. Another six occupants with MAIS score ≥2 injuries had correctly routed seatbelts but overly reclined seat backs, in some cases where the seat back was nearly horizontal. We concluded that this seat-back position had significantly negative effects due to the seatbelt being loosely positioned across the body, thereby allowing excessive forward and sideways motions during rapid MV deceleration. All of the occupants directly impacted the MV interior. One-third of the occupant-related errors were insecure objects in the rear seat that either directly hit six of the drivers, or hit and displaced their seat backs, thereby increasing the seatbelt loading and occupant injuries.

Furthermore, we found that in 13 cases the MVs were older passenger cars lacking side airbags and side curtain airbags, which probably contributed to the injury severity in side impacts. Such airbags help mitigate impacts to the head, chest, abdomen, and pelvis since they provide coverage of the A-pillar, B-pillar, and side roof rail, and further serve as a containment barrier to prevent partial or complete ejection from the MV [33]. Moreover, older MVs also do not include modern crash-avoidance systems, and 81% of the MVs in the current study were older than 10 years. Høye [37] reported that older MVs in Norway are overrepresented in speeding and DUI crashes, and that male and young drivers are overrepresented in these types of crashes. Young adults are more likely to purchase older MVs since they are cheaper. Future government campaigns targeting young adults and parents should emphasize the importance of driving a modern MV equipped with adequate safety equipment. This should also include seatbelt reminders in the front and rear seats. Manufacturers should also be encouraged to equip their MVs with seatbelt interlock devices to prevent the vehicle from being started unless the occupants have fastened their seatbelts [34].

It is especially noteworthy that 89% of the MVCs occurred in rural areas in the present study. Previous research has also found higher rates of MVC fatalities in nonurban environments [35]. Possible contributory factors such as longer travel distances for rural drivers, more-lax attitudes toward MV safety measures, worse road safety (e.g., no barriers between opposing lanes), higher speed limits, greater alcohol consumption, and longer times to receive medical attention have been highlighted [35, 36].

### Strengths and limitations

In-depth investigations of exterior and interior environments of the involved MVs provide far more information about occupant-related factors [37]. However, detailed multidisciplinary studies of real-world MVCs are challenging [9]. We were unable to confirm whether all of the occupants who received MAIS score ≥2 injuries were admitted to hospitals, although we consider this highly likely since occupants with serious injuries would be transported to a hospital, in which case we would have been alerted. One major strength of this study was that experienced collision investigators systematically and prospectively performed the on-scene collection of collision data.

Data obtained from various sources were compared and reviewed by a multidisciplinary team, including assessing seatbelt use or misuse at the time of the collision and the potential severity of other safety errors. The in-depth analyses, photographs, reconstructions of each occupant’s movements inside the MV, and knowledge about the occupants’ injuries provided us with essential detailed data on the factors relevant to MVCs for determining the injury mechanisms. To reduce errors and irregular assessments, data obtained from different sources were compared and reviewed by the same multidisciplinary team. Moreover, data were collected by the same collision investigators, and injury severity scoring was performed in a uniform manner, which should also have reduced the interrater variability. We consider this approach superior to utilizing existing databases.

The small sample of injured occupants with seatbelt misuse, driving with a reclined seat back, or the lack of protective side airbags or side curtain airbags is probably a limitation for identifying these factors as being associated with MAIS score ≥2 injuries. However, the design of the study made us to discover that the injury consequences of such safety errors were severe, and sometimes life threatening.

One limitation of this study is that information about speed and DUI is not always based on exact measurements, and not all occupants involved in the included MVCs were tested for alcohol and drugs. The prevalence of DUI may therefore have been underestimated.

## Conclusion

This study has provided a detailed description of MVCs involving young adult MV occupants, and documented that safety errors and missing safety equipment in the MVs were present in 59% of the cases when the occupants sustained MAIS score ≥2 injuries on Norwegian roads. Our results show that these safety errors are easily avoidable and hence that the ambition of zero young people being killed or seriously injured in MVCs seems achievable. Future work should continue focusing on educational programs for young adults, promoting their awareness about correct seatbelt use, and underlining the importance of driving MVs with up-to-date active and passive safety equipment.

Alcohol consumption, high speeds, and nighttime driving continue to make significant contributions to injuries and fatalities. Future government campaigns should identify and specifically target young adult subgroups such as those who take extra risks or are sensation seekers or recidivist traffic violators. Restrictive measures such as MV impoundment, nonoverridable intelligent speed adaptation, and alcohol interlock devices may be promising measures in the future [37].

## Key points

1. The prevalence of young occupants not using a seatbelt continues to be high and has a negative impact on morbidity and mortality in a significant number of MVCs.
2. Safety errors such as seatbelt misuse and improper sitting position are not uncommon and are likely to be an underestimated factor contributing to severe injuries.
3. Most MVs driven by young adults are older and do not have adequate passive safety equipment.

## Supplementary Information

Below is the link to the electronic supplementary material.

Supplementary file1 (DOCX 37 kb)

## Data Availability

The data will not be deposited.
