# Supplementary material for: Young adults in motor vehicle collisions in Norway: user safety errors observed in majority of cases with severe or fatal injuries
Source: Forensic Sci Med Pathol. 2022 Oct 17;18(4):456–69. doi: 10.1007/s12024-022-00541-x (PMC9636123; doi:10.1007/s12024-022-00541-x)
Supplement: Supplementary file 1 — Supplementary file1 (DOCX 37 kb) [file 12024_2022_541_MOESM1_ESM.docx]

Appendix. Detailed description of the safety errors or missing safety equipment that most likely contributed to the injuries that were detected in 59% (65/111) of the young adult occupants with AIS score ≥ 2 injuries.

Appendix Figure 1: Characteristics of the AIS score ≥ 2-injured young adult occupants with preventable safety errors. Unrestrained occupants (*n* = 33).

Appendix Figure 2: Characteristics of the AIS score ≥ 2-injured young adult occupants with preventable safety errors. Seatbelt misuse: occupants with improper occupant posture relative to seatbelt (*n* = 7).

Appendix Figure 3: Characteristics of the AIS score ≥ 2-injured young adult occupants with preventable safety errors. Seatbelt misuse: occupants with incorrect routing of seatbelt (*n* = 6).

**Appendix Figure 4:** Characteristics of the AIS score ≥ 2-injured young adult occupants with preventable safety errors. Insecure objects/unrestrained passengers in the rear (*n* = 8).

**Appendix Figure 5:** Characteristics of the AIS score ≥ 2-injured young adult occupants with preventable safety errors. Occupants with older MVs without proper passive safety equipment (*n* = 11).

Appendix Figure 1: Characteristics of the AIS score ≥ 2-injured young adult occupants with preventable safety errors. Unrestrained occupants (*n* = 33).

| **Make, model and type of MV** | **First point of impact** | **MV-type of crash partner** | **MV age** | **Delta V (km/h)** | **Intrusion (cm)** | **Sitting location within the MV** | **Toxicology** | **Injuries sustained** | **Injury mechanism** | **Safety errors** | **AIS score** | **ISS** | **NISS** | **Died of injuries** |
| --- | --- | --- | --- | --- | --- | --- | --- | --- | --- | --- | --- | --- | --- | --- |
| VW stw (2004) | Front | Single MVC: fixed object | 11 | 140 | 218 | D | Probably sober | Head/face, chest and abdomen with severe crush injuries. | Unrestrained: head and chest impacted the steering wheel. | Unrestrained | 6 | 75 | 75 | Yes |
| Volvo stw (1963) | Front | Single MVC: fixed object | 40 | 39 | 33 | D | Sober | Heart rupture, hemothorax, rib fractures, bilateral lung contusions. | Unrestrained: chest impacted the steering wheel. | Unrestrained | 6 | 75 | 75 | Yes |
| Audi SUV (2010) | Front | Truck/bus | 3 | 108 | 140 | D | Probably sober | Head crushed, aortic rupture, heart rupture, liver rupture, spleen laceration, kidney laceration. | Unrestrained: head, chest, and abdomen impacted the steering wheel. | Unrestrained | 6 | 75 | 75 | Yes |
| BMW sedan (1998) | Rollover | Single MVC: fixed object | 17 | 21 | 4 | D | Alcohol only | Head crushed, lung contusions. | Unrestrained: head impacted side door window and the head was crushed between MV and the ground during rollover. | Unrestrained | 6 | 75 | 75 | Yes |
| VW stw (2005) | Front | Single MVC: fixed object | 10 | 60 | 65 | D | Sober | Brain contusion, brain herniation and edema, pelvic fracture. | Unrestrained: head and chest, impacted the dashboard and A-pillar. | Unrestrained | 5 | 75 | 75 | Yes |
| Toyota stw (1997) | Rear | SUV/van | 18 | 58 | 135 | D | Sober | Pneumothorax, rib fractures, hemothorax, bilateral lung contusions, diaphragm rupture, ventricle herniation, liver laceration. | Unrestrained: chest and abdomen impacted the steering wheel. | Unrestrained | 5 | 45 | 45 | Yes |
| Opel stw (1997) | Front/side | Truck/bus | 18 | 94 | Measurements not possible | F | Sober | DAI, skull base fractures, bilateral pneumothorax, lung contusion, rib fractures, pelvic fractures, femur fracture, radial fracture. | Unrestrained: head and chest impacted the roof/side door/A-pillar. | Unrestrained.  Olderer MV that lacked side airbag and side curtain airbag | 4 | 41 | 48 | Yes |
| Audi stw (1998) | Front | Truck/bus | 16 | 90 | 74 | F | Illicit drugs only | Brain stem contusion, C1-dislocation, rib fractures, bilateral lung contusions, radial and ulnar fracture. | Unrestrained: head and chest impacted the windshield and dashboard. | Unrestrained | 5 | 33 | 33 | Yes |
| Mercedes sedan (1994) | Rollover | Single MVC: fixed object | 19 | 33 | 41 | F | Sober | Skull fracture, aortic dissection, rib fractures, Th5-6 fracture (no spinal cord injury), spleen rupture, kidney laceration. | Unrestrained: head and chest impacted co-occupant. | Unrestrained | 4 | 29 | 41 | Yes |
| Saab stw (1997) | Rollover | Single MVC: fixed object | 17 | 58 | 60 | D | Alcohol only | Head contusion, t-SAH, DAI gr 2, fracture of Th 3-4 with complete spinal cord injury, rib fractures, bilateral lung contusions, spleen rupture, kidney rupture. | Unrestrained: head impacted side door/roof. | Unrestrained | 5 | 59 | 66 | No |
| VW stw (1998) | Rollover | Single MVC: fixed object | 16 | 50 | 28 | D | Sober | t-SDH, t-SAH, brain contusion, brainstem contusion, skull base fracture, bilateral lung contusions, liver rupture. | Unrestrained; head impacted the roof. | Unrestrained | 5 | 42 | 57 | No |
| VW SUV (2012) | Side | Single MVC: fixed object | 3 | 50 | 35 | F | Sober | Head contusion, DAI gr 3, mandible fracture, facial fracture, rib fractures, bilateral pneumothorax. | Unrestrained; head and chest impacted the side door/roof/A-pillar. | Unrestrained | 5 | 31 | 31 | No |
| BMW sedan (1999) | Side | Single MVC: fixed object | 15 | 50 | 65 | D | Sober | Pelvic fracture. | Unrestrained; pelvic impacted the side door | Unrestrained | 4 | 16 | 16 | No |
| Ford SUV (2006) | Front | Single MVC: fixed object | 7 | 45 | 25 | D | Sober | Dens fracture, C1 fracture, lung contusions, rib fractures. | Unrestrained; head impacted the roof, chest impacted the dashboard. | Unrestrained | 3 | 14 | 17 | No |
| VW stw (2013) | Side | Single MVC: fixed object | 2 | 29 | 10 | F | Sober | Bilateral lung contusions, rib fractures, fracture L1-L5 (no spinal cord injury) | Unrestrained; chest impacted the side door. | Unrestrained | 3 | 14 | 22 | No |
| VW sedan (1994) | Front | Single MVC: fixed object | 21 | 40 | 25 | D | Sober | Femur fracture. | Unrestrained; leg impacted dashboard. | Unrestrained | 3 | 10 | 10 | No |
| Mercedes stw (2013) | Front | Single MVC: fixed object | 1 | 50 | 32 | RR | Alcohol only | Small dislocation of dens axis (no spinal injury), brain contusion. | Unrestrained; head impacted the seatback in front. | Unrestrained | 3 | 10 | 10 | No |
| VW stw (2009) | Front | Single MVC: fixed object | 5 | 68 | 45 | D | Sober | LeFort 2 facial fracture, pelvic fracture. | Unrestrained; face impacted the windshield, chest impacted the steering wheel. | Unrestrained | 2 | 9 | 9 | No |
| VW sedan (1994) | Front | Single MVC: fixed object | 21 | 40 | 25 | F | Sober | Facial fractures, hand fracture. | Unrestrained; face impacted the .windshield | Unrestrained | 2 | 9 | 9 | No |
| BMW stw (2001) | Side | Single MVC: fixed object | 12 | 60 | 80 | RR | Sober | Lung contusion (right side), scapula fracture (right side) | Unrestrained; chest and right arm impacted the side door. | Unrestrained and older MV that lacked side airbag and side curtain airbag in the rear | 2 | 8 | 8 | No |
| Audi stw (2001) | Rollover | Single MVC: fixed object | 13 | 61 | 68 | D | Alcohol only | Pneumothorax, L1-L4 fracture (no spinal cord injury). | Unrestrained: chest in direct contact with MV-interior. | Unrestrained | 2 | 8 | 8 | No |
| Mercedes stw (2013) | Front | Single MVC: fixed object | 1 | 50 | 32 | F | Suspicion of alcohol and/or drugs, not tested | Facial fractures. | Unrestrained; face in contact with windshield. | Unrestrained | 2 | 6 | 6 | No |
| VW sedsan (1994) | Front | Single MVC: fixed object | 21 | 40 | 25 | RR | Sober | Radial fracture, leg fracture, foot fracture. | Unrestrained; arms and legs impacted the seatback in front. | Unrestrained | 2 | 5 | 5 | No |
| Toyota stw (2007) | Front | Stw/sedan | 7 | 68 | 80 | D | Alcohol only | Facial fracture, bilateral pneumothorax. | Unrestrained; face and chest impacted the steering wheel. | Unrestrained | 2 | 5 | 5 | No |
| Opel stw (1997) | Front | Single MVC: fixed object | 16 | 86 | 20 | D | Suspicion of alcohol and/or drugs, not tested | Head contusion, commotio cerebri. | Unrestrained; head impacted the side door on the opposite side. | Unrestrained | 2 | 5 | 5 | No |
| BMW stw (2009) | Rear | Single MVC: fixed object | 4 | 50 | 31 | D | Alcohol only | Head contusion, commotio cerebri. | Unrestrained; head impacted the steering wheel. | Unrestrained | 2 | 5 | 5 | No |
| VW minivan (2005) | Rollover | Single MVC: fixed object | 10 | 40 | 11 | F | Alcohol only | Clavicle fracture, face contusion. | Unrestrained; head and chest impacted the MV-interior. | Unrestrained | 2 | 5 | 5 | No |
| Toyota minivan (2006) | Rollover | Single MVC: fixed object | 9 | 12 | 18 | F | Alcohol only | Rib fractures. | Unrestrained; chest impacted the side door. | Unrestrained | 2 | 5 | 5 | No |
| Suzuki stw (1997) | Rear | Truck/bus | 16 | 87 | 119 | F | Sober | Several vertebral fractures (no spinal cord injuries). | Unrestrained and the passenger seat was displaced forwards due to heavy insecure objects in the rear. | Unrestrained and insecure heavy objects in the rear seat | 2 | 4 | 12 | No |
| Mercedes stw (1990) | Front | Single MVC: fixed object | 24 | 33 | 46 | F | Sober | Humerus fracture. | Unrestrained; arm impacted the side door | Unrestrained and older MV that lacked front airbags, side airbag, and side curtain airbag | 2 | 4 | 1 | No |
| Ford SUV (2006) | Front | Single MVC: fixed object | 7 | 45 | 25 | F | Sober | Lung contusions, clavicle fracture. | Unrestrained; chest impacted the dashboard. | Unrestrained | 2 | 4 | 6 | No |
| VW stw (1997) | Rollover | Single MVC: fixed object | 17 | 50 | 10 | F | Alcohol only | Head contusion, commotio cerebri. | Unrestrained; head impacted the side door. | Unrestrained | 2 | 4 | 4 | No |
| BMW sedan (1999) | Side | Single MVC: fixed object | 15 | 50 | 65 | F | Sober | Shoulder luxation. | Unrestrained; shoulder impacted the steering wheel. | Unrestrained | 2 | 4 | 4 | No |
| MVC = motor vehicle collisions; stw = station wagon; D= driver; F = front seat passenger; LR = left rear passenger; RR = right rear passenger; AIS score = Abbreviated Injury Severity score; ISS = Injury Severity Score, SB = seatbelt; SAH = subarachnoid hemorrhage; DAI = diffuse axonal injury | | | | | | | | | | | | | | |

Appendix Figure 2: Characteristics of the AIS score ≥ 2-injured young adult occupants with preventable safety errors. Seatbelt misuse: occupants with improper occupant posture relative to seatbelt (*n* = 7).

| **Make, model and type of MV** | **First point of impact** | **MV-type of crash partner** | **MV age** | **Delta V (km/h)** | **Intrusion (cm)** | **Sitting location within the MV** | **Toxicology** | **Injuries sustained** | **Injury mechanism** | **Safety errors** | **AIS score** | **ISS** | **NISS** | **Died of injuries** |
| --- | --- | --- | --- | --- | --- | --- | --- | --- | --- | --- | --- | --- | --- | --- |
| Toyota sedan (2008) | Rollover | Single MVC: fixed object | 6 | 50 | 6 | F | Alcohol & illicit drugs | Head completely crushed, liver laceration, right humerus, right femur, right humerus, right tibia and right fibula fracture. | Improper sitting position lead to forward flexion of head and chest and a direct impact with the roof. | Improper occupant posture relative to SB due to reclined sitting position. | 6 | 75 | 75 | Yes |
| Audi stw (1997) | Front | Single MVC: fixed object | 18 | 60 | 126 | F | Sober | Brain contusions, t-SAH, skull base fracture, bilateral lung contusions, hemothorax, liver contusion, radial, ulnar, and pelvic fracture. | Reclined sitting position (seatback in too much of a rearward position) caused excessive forward flexion of head and chest and impact with the A-pillar and dashboard. | Improper occupant posture relative to SB due to reclined sitting position. | 4 | 39 | 36 | No |
| VW stw (1999) | Front | Single MVC: fixed object | 14 | 50 | 146 | F | Alcohol only | Head contusion, t-SDH, DAI gr 3. | Reclined sitting position (seatback in too much of a rearward position) caused excessive forward flexion of head and impact with the dashboard. | Improper occupant posture relative to SB due to reclined sitting position. | 4 | 17 | 26 | No |
| Citroen stw (2010) | Front/side | Single MVC: fixed object | 3 | 50 | 100 | D | Sober | Pneumothorax, pelvic fracture, femur fracture. | Chest and pelvis impacted the side door/A-pillar. | Improper occupant posture relative to SB due to reclined.  Sitting position and older MV that lacked side airbag curtains | 3 | 14 | 17 | No |
| Toyota stw (2004) | Front | SUV/van | 10 | 47 | 52 | LR | Sober | Lung contusions, vertebra fracture (TH 12; no spinal cord injury), radial and ulnar fracture. | Reclined sitting position (seatback in too much of a rearward position) caused excessive forward flexion of chest, impact with seatback in front. | Improper occupant posture due to reclined sitting position. | 3 | 14 | 14 | No |
| Mercedes sedan (1993) | Front | Single MVC: fixed object | 22 | 29 | 26 | F | Sober | Chest contusion, compression fracture Th 12 (no spinal cord injury). | Reclined sitting position (seatback in too much of a rearward position) caused excessive chest movement and increased load on the spine. | Improper occupant posture due to reclined sitting position. | 2 | 5 | 5 | No |
| Audi stw (2005) | Front | Single MVC: fixed object | 10 | 50 | 28 | F | Sober | Chance fracture Th12 (no spinal cord injury). | Reclined sitting position (seatback in too much of a rearward position) caused increased movement the torso and impact with the center console. | Improper occupant posture due to reclined sitting position. | 2 | 5 | 5 | No |
| MVC = motor vehicle collisions; stw = station wagon; D= driver; F = front seat passenger; LR = left rear passenger; RR = right rear passenger; AIS score = Abbreviated Injury Severity score; ISS = Injury Severity Score, SB = seatbelt; SAH = subarachnoid hemorrhage; DAI = diffuse axonal injury | | | | | | | | | | | | | | |

Appendix Figure 3: Characteristics of the AIS score ≥ 2-injured young adult occupants with preventable safety errors. Seatbelt misuse: occupants with incorrect routing of seatbelt (*n* = 6).

| **Make, model and type of MV** | **First point of impact** | **MV-type of crash partner** | **MV age** | **Delta V (km/h)** | **Intrusion (cm)** | **Sitting location within the MV** | **Toxicology** | **Injuries sustained** | **Injury mechanism** | **Safety errors** | **AIS score** | **ISS** | **NISS** | **Died of injuries** |
| --- | --- | --- | --- | --- | --- | --- | --- | --- | --- | --- | --- | --- | --- | --- |
| Audi stw (2008) | Side | Truck/bus | 6 | 65 | 33 | LR | Sober | Head contusion, brain contusion, C1/C2 ligament injury, t-SAH, facial fractures, rib fractures, milt rupture. | SB misuse (shoulder part of SB under arm) lead to forward flexion of head and chest and impact with the driver seatback. | SB misuse with SB under arm | 5 | 59 | 59 | Yes |
| Audi stw (1997) | Front | Single MVC: fixed object | 18 | 60 | 28 | RR | Probably sober | t-SAH, t-SDH, brain contusion. | SB misuse (shoulder part of SB under arm) caused excessive forward flexion of head and impact with the seatback in front. | SB misuse with SB under arm | 5 | 25 | 25 | Yes |
| Skoda stw (2005) | Front | Stw/sedan | 7 | 68 | 65 | RR | Sober | Rupture of diaphragm, ventricle herniation, hemothorax, rib fractures, bilateral lung contusions, intestine perforation, liver laceration, SB sign and decollement of skin on abdomen. | SB misuse, shoulder part of SB under right arm, increased SB-loading on thorax and abdomen as well as impact with the seatback in front. | SB misuse with SB under arm | 4 | 29 | 29 | No |
| Ford stw (2014) | Front | Single MVC: fixed object | 0 | 60 | 10 | CR | Sober | Liver laceration, vertebra fracture (Th 12; no spinal cord injury), SB sign chest and abdomen. | SB misuse, shoulder part of SB under thes right arm, increased SB-loading on chest and abdomen. | SB misuse with SB under arm | 3 | 9 | 14 | No |
| Volvo SUV (2014) | Front | Stw/sedan | 1 | 54 | 70 | LR | Sober | Compression fracture Th6 and L4 (no spinal cord injuries). | SB misuse (SB too high on abdomen) and insecure heavy objects in the trunk displaced the rear seat >10 cm forwards. | SB misuse with SB too high on abdomen and heavy insecure objects in the trunk | 2 | 9 | 9 | No |
| BMW sedan (1998) | Rollover | Single MVC: fixed object | 15 | 36 | 18 | D | Sober | Head contusion, commotio cerebri. | SB misuse, SB under left arm, increased excessive movement of head and impact with A-pillar. | SB misuse with SB under arm and improper occupant posture due to reclined sitting position | 2 | 4 | 4 | No |
| MVC = motor vehicle collisions; stw = station wagon; D= driver; F = front seat passenger; LR = left rear passenger; RR = right rear passenger; AIS score = Abbreviated Injury Severity score; ISS = Injury Severity Score, SB = seatbelt; SAH = subarachnoid hemorrhage; DAI = diffuse axonal injury | | | | | | | | | | | | | | |

Appendix Figure 4: Characteristics of the AIS score ≥ 2-injured young adult occupants with preventable safety errors. Insecure objects/unrestrained passengers in the rear (*n* = 8).

| **Make, model and type of MV** | **First point of impact** | **MV-type of crash partner** | **MV age** | **Delta V (km/h)** | **Intrusion (cm)** | **Sitting location within the MV** | **Toxicology** | **Injuries sustained** | **Injury mechanism** | **Safety errors** | **AIS score** | **ISS** | **NISS** | **Died of injuries** |
| --- | --- | --- | --- | --- | --- | --- | --- | --- | --- | --- | --- | --- | --- | --- |
| Peugot minivan (2006) | Front | Truck/bus | 9 | 86 | 25 | D | Illicit drugs only | Skull base fracture, facial fracture, brain contusion, bilateral lung contusions, rib fractures, pneumothorax, right radial and femur fracture. | Insecure heavy cargo in the rear displacing the driver seat forwards. Head and chest impacted the steering wheel. | Insecure heavy objects in the rear seat displacing the driver seat forwards. | 5 | 50 | 50 | Yes |
| Volvo stw (1995) | Side | Stw/sedan | 18 | 108 | 90 | D | Sober | Head contusion, brain edema, C1-luxation, bilateral lung contusions, bilateral pneumothorax, flail chest, spleen contusion, small intestine contusion, pelvic fracture. | Heavy insecure cargo impacted the head and chest as well as torso in direct contact with the center console and dashboard. | Insecure heavy cargo in the rear seat. | 5 | 50 | 50 | Yes |
| VW sedan (1994) | Front | Truck/bus | 21 | 94 | 152 | D | Sober | Skull base fracture, facial fractures, ulnar, femur, tibia fracture. | Heavy cargo displaced driver seat >20 cm forward. Head impacted with A-pillar, left arm and leg in contact with left side door/steering wheel. | Insecure heavy objects in the rear seat displacing the driver seat forwards. | 3 | 19 | 22 | Yes |
| Opel stw (1999) | Front | Stw/sedan | 24 | 54 | 50 | D | Sober | Left femur, tibia, foot fracture. | Insecure heavy cargo in the rear seat displacing the driver seat forwards. Legs impacted the dashboard. | Insecure heavy objects in the rear seat displacing the driver seat forwards. | 3 | 10 | 10 | No |
| VW stw (1990) | Front | Stw/sedan | 23 | 21 | 90 | D | Sober | Facial fractures, hand and foot fractures. | Insecure heavy insecure cargo in the rear seat. Head impacted the steering wheel, hand hit dashboard and feet hit pedals. | Insecure heavy objects in the rear-seat probably displacing the driver seat forwards. | 2 | 6 | 6 | No |
| VW stw (2003) | Rollover | Single MVC: fixed object | 11 | 60 | 38 | D | Alcohol only | Head contusion, pneumothorax. | Head and chest hit by heavy insecure cargo. | Insecure heavy objects in the rear seat. | 2 | 2 | 5 | No |
|  | | | | | | | | | | | | | | |
| Audi stw (1997) | Front | Single MVC: fixed object | 18 | 60 | 28 | D | Probably sober | Head/facial contusions, facial fractures, ankle fracture. | Unrestrained rear-seat passenger displaced the front seatback > 20 cm. Excessive forward flexion of head and direct impact with steering wheel. | Unrestrained rear-seat passenger displacing the driver seat forwards. | 2 | 9 | 9 | No |
| Audi stw (2008) | Side | Truck/bus | 6 | 65 | 33 | D | Sober | Lung contusions, small heart contusion. | Excessive forward flexion of head and direct impact with steering wheel/dashboard, unrestrained rear-seat passenger displaced the front seatback > 20 cm. | Unrestrained rear-seat passenger displacing the driver seat forwards. | 2 | 5 | 5 | No |
| MVC = motor vehicle collisions; stw = station wagon; D= driver; F = front seat passenger; LR = left rear passenger; RR = right rear passenger; AIS score = Abbreviated Injury Severity score, ISS = Injury Severity Score, SB = seatbelt; SAH = subarachnoid hemorrhage; DAI = diffuse axonal injury | | | | | | | | | | | | | | |

**Appendix Figure** **5**: Characteristics of the AIS **score** ≥ 2-injured young adult occupants with preventable safety errors. Occupants with older MVs without proper passive safety equipment (*n* = 11).

| **Make, model and type of MV** | **First point of impact** | **MV-type of crash partner** | **MV age** | **Delta V (km/h)** | **Intrusion (cm)** | **Sitting location within the MV** | **Toxicology** | **Injuries sustained** | **Injury mechanism** | **Safety errors** | **AIS score** | **ISS** | **NISS** | **Died of injuries** |
| --- | --- | --- | --- | --- | --- | --- | --- | --- | --- | --- | --- | --- | --- | --- |
| Volvo stw (1994) | Side | Single MVC: fixed object | 21 | 61 | 68 | D | Sober | Head contusion, severe burn injuries. | Head impacted the side door/roof. | Older MV that lacked side airbag and side curtain airbag. | 6 | 75 | 75 | Yes |
| Volvo stw (1995) | Side | Stw/sedan | 18 | 83 | 90 | F | Sober | Facial fractures, aortic rupture, rib fractures, diaphragm rupture, bilateral lung contusions, spleen rupture, liver rupture, intestine contusion, fracture of the humerus, femur, and pelvis. | Head impacted the roof, chest and abdomen impacted the side door. | Older MV that lacked side airbag and side curtain airbag. | 5 | 50 | 57 | Yes |
| VW stw (1998) | Front | Single MVC: fixed object | 17 | 79 | 51 | D | Alcohol only | Bilateral pneumothorax, bilateral lung contusions, brain contusion, spleen contusion, liver contusion, radial, femur, and scapula fracture. | Head impacted the roof due to intrusion of vehicle interior, chest and abdomen in contact with steering wheel/dashboard. | Older MV that lacked side airbag and side curtain airbag. | 4 | 38 | 38 | No |
| Opel stw (2000) | Rollover | Single MVC: fixed object | 15 | 48 | 40 | D | Sober | Fracture of C6 and C7, complete spinal cord injury, radial fracture. | Neck and chest in direct impact with side door/roof and hit by heavy insecure objects in the rear seat. | Older MV that lacked side airbag and side curtain airbag. | 5 | 30 | 30 | No |
| Audi stw (1996) | Rollover | Single MVC: fixed object | 18 | 30 | 56 | LR | Sober | Skull base fracture, rib fractures. | Head and chest impacted roof/side door. | Older MV that lacked side airbag and side curtain airbag. | 3 | 18 | 18 | No |
| Mazda stw (1994) | Side | Single MVC: fixed object | 17 | 61 | 50 | F | Sober | Facial laceration, pelvic fracture. | Head impacted the roof and the pelvis struck the center console. | Older MV that lacked side airbag and side curtain airbag. | 3 | 11 | 11 | No |
| Ford sedan (1979) | Front | Single MVC: fixed object | 36 | 50 | 30 | D | Alcohol only | Head contusion, femur fracture. | Head and thigh impacted the side door. | Older MV that lacked side airbag and side curtain airbag. | 3 | 10 | 10 | No |
| Mercedes stw (1991) | Side | Single MVC: fixed object | 22 | 50 | 72 | D | Sober | Rib fractures, pneumothorax, small liver contusion, vertebral fractures L2-L5 (no spinal cord injury). | Chest and abdomen impacted the side door/steering wheel and center console. | Older MV that lacked side airbag and side curtain airbag. | 2 | 10 | 10 | No |
| Mazda stw (1994) | Side | Single MVC: fixed object | 17 | 61 | 50 | D | Sober | Head contusion, pelvic fracture. | Head impacted the side door/roof and steering wheel. | Older MV that lacked side airbag and side curtain airbag. | 2 | 8 | 8 | No |
| Toyota minivan (2001) | Rollover | Single MVC: fixed object | 14 | 18 | 23 | D | Sober | Head contusion, brief loss of consciousness. | Head impacted the roof/side door. | Older MV that lacked side airbag and side curtain airbag. | 2 | 5 | 5 | No |
| Audi stw (1996) | Rollover | Single MVC: fixed object | 18 | 30 | 56 | D | Suspicion of alcohol and/or drugs, not tested | Head contusion, brief loss of consciousness. | Head impacted the B-pillar/side door. | Older MV that lacked side airbag and side curtain airbag. | 2 | 5 | 5 | No |
| MVC = motor vehicle collisions; stw = station wagon; D= driver; F = front seat passenger; LR = left rear passenger; RR = right rear passenger; AIS score = Abbreviated Injury Severity score; ISS = Injury Severity Score, SB = seatbelt; SAH = subarachnoid hemorrhage; DAI = diffuse axonal injury | | | | | | | | | | | | | | |
